# Craniofacial and dental toxicity of dioxins: developmental phenotypes and mechanistic insights

**DOI:** 10.3389/fcell.2026.1878726

**Published:** 2026-08-19

**Authors:** Mohammadreza Vatankhah, Jian Xu

**Affiliations:** Center for Craniofacial Molecular Biology, Department of Biomedical Sciences, Herman Ostrow School of Dentistry, University of Southern California, Los Angeles, CA, United States

**Keywords:** 2,3,7,8-Tetrachlorodibenzo-p-dioxin (TCDD), aryl hydrocarbon receptor (AhR), cleft palate, craniofacial development, developmental toxicity, environmental exposure, odontogenesis, palatogenesis

## Abstract

**Background:**

Dioxins are persistent environmental pollutants with high lipophilicity and long half-lives, enabling their accumulation in biological tissues and ecosystems. Among them, 2,3,7,8-tetrachlorodibenzo-p-dioxin (TCDD) is the most toxic and extensively studied chemical. Human exposure occurs primarily through contaminated food, cigarette smoke, and industrial emissions, posing significant health risks.

**Objective:**

This review summarizes the effects of dioxins, particularly TCDD, on craniofacial and dental development and discusses recent findings on the mechanisms underlying these effects.

**Methods:**

A narrative review of experimental studies in animal models, *in vitro* systems, and human study data was conducted to evaluate phenotypic and mechanistic outcomes of TCDD exposure across palatogenesis, craniofacial bone formation, and tooth development.

**Results:**

In the developing palate, TCDD impairs mesenchymal proliferation, shelf elevation, and fusion, resulting in cleft palate involving various mechanisms such as oxidative stress, apoptosis, epigenetic changes, and suppression of TGF-β and Wnt signaling. Craniofacial bone development is also affected, with reduced ossification, altered mineralization, and impaired osteoblast differentiation. These outcomes are largely mediated by aberrant aryl hydrocarbon receptor (AhR) activation. However, other pathways, such as retinoic acid and vitamin D signaling, have also been found to have roles in these outcomes. Furthermore, genetic background modulates susceptibility, as shown by strain-specific differences and the protective role of AhR repressors. TCDD disrupts odontogenesis in a stage- and dose-dependent manner, leading to enamel hypoplasia, molar agenesis, dentin defects, and increased caries susceptibility.

**Conclusion:**

Dioxins, particularly TCDD, are potent developmental toxicants affecting multiple craniofacial tissues. Their effects are driven by dysregulation of several critical signaling pathways, with AhR acting as the central mediator. Given their long half-life and bioaccumulation, dioxins remain a relevant public health threat.

## 1 Introduction

Environmental factors, including chemical components in cigarette smoke, food, and pollution, have long been recognized to correlate with congenital defects (Baldacci et al., 2018). One of the most common areas where these defects occur is the craniofacial region, including the palate, skull, and dental structures. Craniofacial development involves the migration and differentiation of neural crest and mesoderm-derived cells, and coordinated growth and fusion leading to the formation of the face and skull (Kang and Svoboda, 2005; Beames and Lipinski, 2020). This complex process is influenced by both genetic and environmental factors (Edison and Muenke, 2003; Beames and Lipinski, 2020; Al-Obaidi et al., 2025). An understanding of gene-environment crosstalk would lay the foundation for potential preventive and therapeutic measures.

Chemical components of environmental exposures such as cigarette smoke and pollution are strongly associated with teratogenic defects (Dolk and Vrijheid, 2003; Baldacci et al., 2018; Leśków et al., 2019). Several distinct chemical classes disrupt skeletal and craniofacial development in particular: heavy metals such as lead and cadmium impair bone mineralization and growth (Oginawati et al., 2022); endocrine-disrupting chemicals including bisphenol A and phthalates alter osteogenic differentiation (Hwang et al., 2013; Zhang et al., 2024b) and persistent organic pollutants such as flame retardants (Yan and Hales, 2019) and dioxins (Hornung et al., 1999; Burns et al., 2015; Sholts et al., 2015a) disrupt bone and cartilage formation. Given the broad diversity of teratogenic chemicals, research has focused on representative groups to identify shared mechanisms, with dioxins being one of the most potent and widely studied groups. Dioxins are a class of chemicals that are mostly produced through industrial processes, including incineration, paper bleaching, and the production of pesticides (Kulkarni et al., 2008). These processes release dioxins into the environment, where they can enter the food chain. Humans are mainly exposed to dioxins through contaminated food and living in the vicinity of industrial facilities (Demond et al., 2012; Fernandes and Falandysz, 2021). Moreover, dioxins are one of the most common chemicals found and studied in cigarette smoke (Muto and Takizawa, 1989; Dertinger et al., 2001; Kasai et al., 2006; Xue et al., 2016). Since dioxins have a significantly high stability and long half-life (Kerger et al., 2006), they will accumulate in tissues over a person’s lifetime, leading to various health risks (Kogevinas, 2001; Hays and Aylward, 2003).

## 2 Dioxins: environmental persistence and implications for human health

Dioxins are formed during processes that involve chlorine-containing compounds and organic matter at high temperatures. Under these circumstances, chlorine radicals can react with carbon-rich materials such as aromatic hydrocarbons or chlorinated phenols. Chlorine atoms substitute hydrogen atoms on these carbon frameworks, and after oxidative coupling, dioxins are formed (Shibamoto et al., 2007). Dioxin compounds share the same basic chemical structure, consisting of two benzene rings connected by two oxygen atoms in a 1,4-dioxin ring system, with chlorine atoms attached to the benzene rings at any of the 8 possible sites. The number and position of these chlorine atoms determine the toxicity of the dioxin (Birnbaum, 1994). The most toxic dioxin compound is 2,3,7,8-Tetrachlorodibenzo-p-dioxin (TCDD), which has four chlorine atoms. TCDD is the *de facto* standard for dioxin studies and, consequently, the most extensively studied compound in this field (Mandal, 2005; Sobol et al., 2024). The toxicity of different dioxins, for comparative purposes, is indicated by the toxic equivalency factor (TEF), with a TEF value assigned to each compound based on its relative toxicity compared with TCDD (Van den Berg et al., 1998; Van den Berg et al., 2006; DeVito et al., 2024). TCDD, as the most toxic dioxin, is assigned the maximum TEF of 1. Other dioxins are scored as fractions of 1, reflecting their lower relative toxicity (Van den Berg et al., 1998; Van den Berg et al., 2006; DeVito et al., 2024).

**FIGURE 1:**
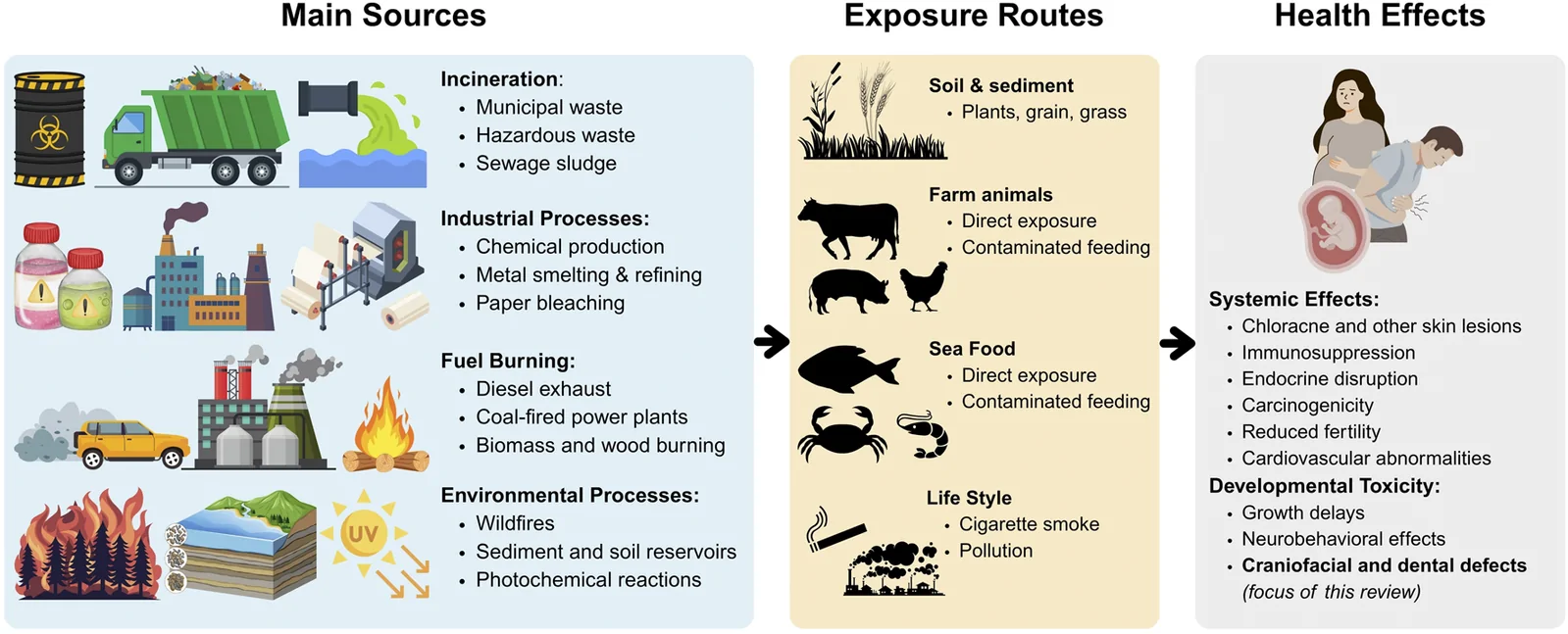
Sources, environmental fate, and health impacts of dioxins. This schematic illustrates the three major components of dioxin contamination: (Left panel) Primary sources of environmental dioxin release including industrial processes (waste incineration, chemical manufacturing, metal smelting, paper bleaching), combustion activities (diesel exhaust, coal-fired power plants, residential wood burning), and natural/environmental processes (wildfires, atmospheric photochemical reactions, and re-release from contaminated sediment and soil reservoirs); (Center panel) Environmental distribution and human exposure pathways showing dioxin accumulation in soil and sediment, atmospheric and aquatic dispersal, bioaccumulation through food chains, and the predominant routes of human exposure via contaminated food (particularly animal products), with additional exposure through soil/dust ingestion and inhalation. Critical exposure windows are highlighted including *in utero* transfer, lactational exposure, and lifetime dietary accumulation; (Right panel) Spectrum of adverse health outcomes associated with dioxin exposure across different life stages: fetal/developmental effects (cleft palate, dental abnormalities, neurodevelopmental disorders), childhood impacts (immunosuppression, endocrine disruption, growth retardation), and adult manifestations (various cancers, cardiovascular disease, reproductive dysfunction, metabolic disorders including type 2 diabetes, and chloracne).

Dioxins are produced through various chemical processes (Figure 1). The largest fraction of dioxin production nowadays includes industrial processes such as waste incineration (municipal, hazardous, and medical), metal smelting and refining, and the production of chlorinated chemicals like pesticides and herbicides (Weber et al., 2008a; Weber et al., 2008b). Inadequate air pollution control strategies in these facilities have historically exacerbated emissions (Dopico and Gómez, 2015). Due to dioxins’ high stability, dioxin contaminations that occurred in the past remain relevant in the present and future. For instance, the impacts of the Leblanc Soda factory, which operated from 1848 to 1893 in Germany and formed toxic compounds equivalent to 1–10 kg of TCDD, are still traceable nowadays (Weber et al., 2008a). Another infamous example of dioxin contamination in the soil resulted from the use of defoliants in the Vietnam War, which released toxic compounds equivalent to 130–366 kg of TCDD (Weber et al., 2008a). After more than 60 years, substantial residual pollution persists in the affected soil (Croes et al., 2011; Van Thuong et al., 2015; My et al., 2021). Another major contamination event occurred in 1976, when an explosion at a chemical factory near Seveso, Italy, exposed residents to TCDD. (Eskenazi et al., 2018; Warner et al., 2020; Eskenazi et al., 2021).

Altogether, dioxins contaminate the soil, ocean, plants, and eventually enter animals, including fish, wild animals, and farm animals. Human exposure to dioxins mainly occurs due to the ingestion of such contaminated food (Travis and Hattemer-Frey, 1991). Foods like meat and dairy products are the primary contributors to human dioxin intake (Fiedler et al., 1990). Another route of exposure to dioxin is through cigarette smoking, which contains a notable concentration of dioxin compounds that activate dioxin-related pathways (Kitamura and Kasai, 2007; Wilson et al., 2008).

Dioxins, especially the tetra- and higher chlorinated congeners, are highly stable (Atkinson, 1991). The only significant degradation process in the environment is photodegradation of non-sorbed species in the gaseous phase (Kulkarni et al., 2008). Hence, dioxins are removed faster in the atmosphere through photolysis and reactions with OH radicals (Atkinson, 1991). However, when in soil, they are highly stable. Their half-life in surface soil is approximately 10–12 years, and deeper, they can stay unchanged for decades (Sinkkonen and Paasivirta, 2000).

As dioxins are highly soluble in fat, liver and adipose tissue are their major storage sites in most mammalian species, with further deposition in the skin and adrenal gland (Van den Berg et al., 1994). Maternal exposure to TCDD, additionally, causes significant accumulation in the embryonic liver and head (Nau and Bass, 1981; Weber and Birnbaum, 1985; Krowke and Neubert, 1990). Human epidemiological studies have linked dioxin exposure to a range of adverse effects, including metabolic syndromes (Uemura et al., 2009; Liang et al., 2020), infertility (Eskenazi et al., 2021), cancer (Xu et al., 2016), and impaired fetal and postnatal growth (Wesselink et al., 2014; Pan et al., 2015; Tai et al., 2016). Evidence from both animal and human studies further associates dioxins with congenital abnormalities such as neurological disorders and dental/craniofacial defects (Alaluusua et al., 2004; Guo et al., 2018).

Human exposure is not to TCDD alone but to a mixture of dioxin-like compounds (polychlorinated dibenzo-p-dioxins (PCDDs), dibenzofurans (PCDFs), and dioxin-like PCBs) that act through a shared aryl hydrocarbon receptor (AhR)-mediated mechanism and are placed on a common scale by the WHO toxic-equivalency (TEF) framework (Van den Berg et al., 2006; DeVito et al., 2024). Within this mixture, TCDD is often a minor contributor to total body-burden TEQ. For instance, dioxin-like PCBs alone accounted for roughly 40% of total TEQ in a biomonitoring cohort (Fromme et al., 2015), with comparable PCDD/F and PCB contributions in other populations (Uemura et al., 2009; Bruce-Vanderpuije et al., 2019). Because these congeners share the AhR-dependent mode of action, animal and cell culture findings established using TCDD as a research tool provide a mechanistic basis for understanding the dioxin class as a whole, while the TEF framework allows their relative potencies to be compared quantitatively. Experimental evidence supports this shared mechanism. Structurally distinct dioxin-like congeners, including polychlorinated dibenzofurans and dioxin-like PCBs, reproduce the craniofacial and dental phenotypes caused by TCDD, and they differ in potency rather than in the type of defect, as discussed in the corresponding sections below. Since these chemicals are all ligands to the AhR, their convergence on the same phenotypes indicates that AhR is the shared mediator of these effects.

Direct human data on dioxin-induced craniofacial and dental defects come largely from high-exposure cohorts. After the 1976 Seveso accident, residents with the highest TCDD body burdens showed developmental dental aberrations such as enamel defects and missing teeth in those exposed during childhood (Alaluusua et al., 2004), alongside altered reproductive and developmental outcomes in later follow-up (Eskenazi et al., 2018; Warner et al., 2020). The Yusho and Yu-Cheng poisonings, although driven by heat-degraded PCBs and mixtures of their PCDF by-products rather than dioxins, produced natal teeth and a broader ectodermal-tissue disorder in prenatally exposed children (Miller, 1985; Yamashita and Hayashi, 1985; Rogan et al., 1988), and dioxin exposure has been associated with cleft palate in epidemiological analysis (Leśków et al., 2019). These observations are associative and involve mixed exposures and small cohorts. The controlled evidence on dose, timing, and mechanism reviewed below therefore comes mainly from animal and *in vitro* models. Since palatogenesis, craniofacial osteogenesis, and odontogenesis are highly conserved between rodents and humans, relying on the same signaling pathways, including TGF-β, Wnt/β-catenin, BMP, FGF, and SHH, these models serve as informative surrogates for human craniofacial and dental development. Experimental doses span a wide range depending on the endpoint: overt teratogenic outcomes such as cleft palate are typically induced at tens to hundreds of µg/kg, whereas sensitive dental endpoints (e.g., third-molar size and cusp morphology) respond to doses as low as 0.01–1.0 μg/kg. Even the lower end exceeds typical human background exposures (on the order of pg–ng/kg per day, corresponding to body burdens of a few pg TEQ per gram of lipid). Even so, accidental and occupational exposures have reached far higher body burdens, more than 25 μg/kg lipid in the Seveso accident, for instance (Kogevinas, 2001; Alaluusua et al., 2004; Eskenazi et al., 2014). These models therefore contribute to hazard identification and to resolving developmental windows and mechanisms, rather than serving as direct quantitative predictors of risk at environmental exposure levels.

In this review, we will summarize the impact of dioxins on craniofacial and dental structures and highlight recent findings that shed light on the underlying molecular mechanisms.

## 3 Effects of dioxin on palatogenesis

**FIGURE 2:**
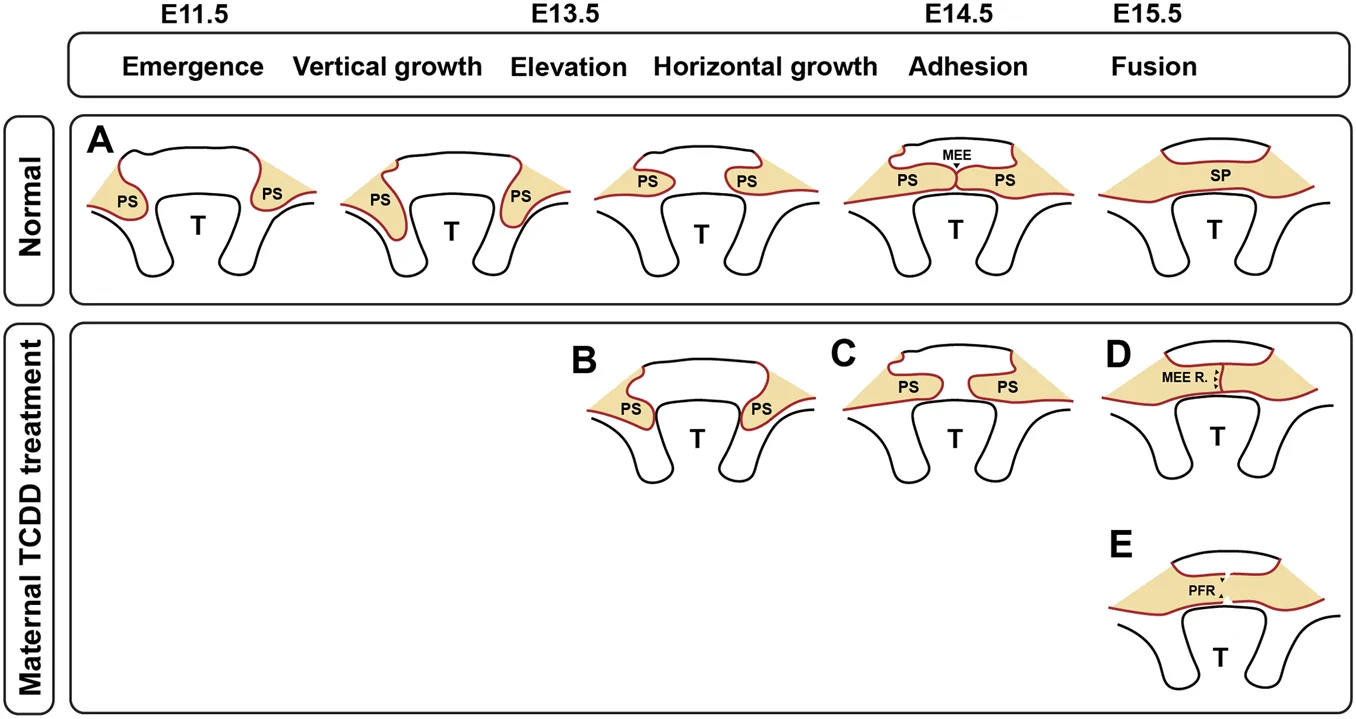
Secondary palate development in normal and TCDD-exposed mouse embryos. **(A)** Normal palatogenesis from E11.5–E15.5 showing sequential developmental stages: palatal shelf (PS) emergence from maxillary processes, vertical growth alongside the tongue (T), elevation above the tongue, horizontal growth toward the midline, adhesion via medial edge epithelium (MEE) contact, and complete fusion following MEE degradation to form the secondary palate (SP). **(B–E)** Maternal TCDD treatment disrupts specific developmental stages: **(B)** Impaired palatal shelf elevation - shelves fail to reorient from vertical to horizontal position, remaining trapped lateral to the tongue. **(C)** Defective horizontal growth and mesenchymal proliferation resulting in hypoplastic shelves unable to reach the midline. **(D)** Persistence of MEE remnants (MEE R.) at the fusion site due to failed epithelial-mesenchymal transition, apoptosis, or epithelial extrusion. **(E)** Post-fusion rupture (PFR) demonstrating pathological separation of initially fused palatal shelves caused by inadequate mesenchymal condensation and incomplete MEE removal.

Palatogenesis, the process of palate formation during embryonic development, is highly conserved between humans and mice. It involves a complex developmental sequence to form the primary and secondary palate. The primary palate arises from the frontonasal prominence and contains the premaxillary region of the jaw, including the upper lip and upper incisor area anterior to the incisive foramen; the secondary palate, formed from the outgrowth of bilateral maxillary prominences, develops into the remaining hard and soft palate structures (Li et al., 2017). Three critical events orchestrate the secondary palatal development: vertical growth of palatal shelves, elevation of the shelves to a horizontal position superior to the dorsum of the tongue, and bilateral fusion at the midline (Bush and Jiang, 2012) (Figure 2). Disruption of these processes at any step(s), by genetic and/or environmental factors, can result in cleft lip and palate or cleft palate (CP) (Bush and Jiang, 2012). CP is the most prevalent human craniofacial birth defect, affecting approximately 1 in 700–2000 newborns (Mossey et al., 2009). In humans, cases of CP have been linked to dioxin exposure (Leśków et al., 2019). In mouse models, dioxins, particularly TCDD, are documented causal toxins for CP (Hassoun et al., 1984; Krowke and Neubert, 1990; Mimura et al., 1997; Ishida et al., 2004; Imura et al., 2010). The teratogenic effects of TCDD on palatogenesis are highly dependent on the timing and dosage of exposure, with the most vulnerable period occurring between E10–E13 in mice, corresponding to the critical stages of palatal shelf growth and elevation. TCDD exposure during this window resulted in a dose-dependent increase in CP incidence, with a nearly 100% occurrence at a dose of 40 μg/kg. Lower doses of TCDD, such as 10 or 20 μg/kg, produced variable outcomes, ranging from incomplete palatal elevation to delayed fusion, indicating that the severity and frequency of CP correlate with TCDD concentration (Yamada et al., 2006). CP is not restricted to TCDD, and other dioxin-like compounds can cause CP as well. In C57BL/6N mice, the polychlorinated dibenzofurans 1-PeCDF, 4-PeCDF, and 1,2,3,4,7,8-HCDF, administered on gestational days 10–13, induce CP and hydronephrosis, the two important characteristics of dioxin-like teratogenicity, with steep dose-response curves parallel to those of TCDD (Birnbaum et al., 1987). Among these congeners, 4-PeCDF is the most teratogenic and also carries the highest TEF, acting at approximately one-tenth the potency of TCDD (Birnbaum et al., 1987). The exposure window and the resulting phenotype are the same as those of TCDD, which indicates that these congeners might share a similar AhR-dependent mechanism.

### 3.1 AhR signaling and genetic susceptibility

**FIGURE 3:**
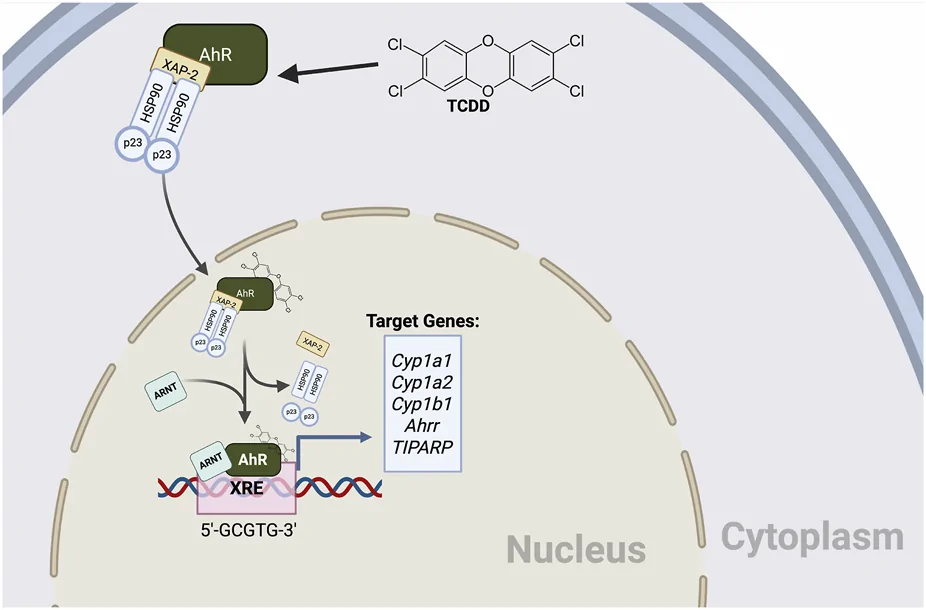
Canonical aryl hydrocarbon receptor (AhR) signaling pathway activated by TCDD. In the absence of ligand, AhR resides in the cytoplasm in a multiprotein chaperone complex containing heat shock protein 90 (HSP90), co-chaperone p23, and immunophilin-like protein XAP-2 (also designated AIP or ARA9), which together stabilize AhR in a high-affinity ligand-binding conformation and prevent premature nuclear import. TCDD diffuses across the plasma membrane and binds the PAS-B domain of AhR, inducing a conformational change that exposes the nuclear localization signal. The ligand-bound AhR translocates into the nucleus, where the chaperone complex (HSP90, p23, XAP-2) dissociates. Nuclear AhR then heterodimerizes with the aryl hydrocarbon receptor nuclear translocator (ARNT). The AhR/ARNT heterodimer binds xenobiotic response elements (XREs, also termed dioxin response elements) bearing the core consensus sequence 5′-GCGTG-3′ in the regulatory regions of target genes, recruiting coactivators and driving transcription of phase I xenobiotic-metabolizing enzymes (*Cyp1a1*, *Cyp1a2*, *Cyp1b1*) and feedback regulators of the pathway, including the AhR repressor (*Ahrr*) and TCDD-inducible poly (ADP-ribose) polymerase (*TIPARP*). Subcellular compartments (cytoplasm and nucleus) and the nuclear envelope are indicated.

TCDD toxicity in palatogenesis is mainly mediated by its receptor, AhR, as *Ahr*-null mice show complete resistance to TCDD-induced CP (Mimura et al., 1997; Peters et al., 1999). AhR is a basic helix-loop-helix–PER-ARNT-SIM transcription factor that is bound by TCDD with high affinity (Baccarelli et al., 2004; Singh et al., 2007). It responds to both exogenous ligands, including dioxins and PAHs, and endogenous ligands, such as tryptophan metabolites and indoxyl sulfate, and activates toxicant metabolism and oxidative stress response (Denison and Nagy, 2003). Upon ligand binding, AhR translocates to the nucleus, dimerizes with the aryl hydrocarbon receptor nuclear translocator (ARNT), and binds to xenobiotic response elements (XREs) found in the promoters, enhancers, and intronic regions of target genes, including cytochrome P450 enzymes (CYP1A1, CYP1B1), which mediate toxicant metabolism and oxidative stress responses (Abbott et al., 1994). In the absence of ligand, AhR is sequestered in the cytoplasm within a chaperone protein complex that includes HSP90, p23 and XAP2. This complex masks the nuclear localization signal (NLS) at the N-terminus of AhR and protects it from proteasomal degradation (Dvořák and Přikryl, 2026) (Figure 3). AhR is widely expressed across craniofacial and skeletal tissues including the palatal mesenchymal cells (Yamada et al., 2014; Liu et al., 2020), palatal epithelium (Yamada et al., 2014), periodontal ligament cells (Tomokiyo et al., 2012), gingival epithelial cells (Engen et al., 2017; Li et al., 2019), and the developing teeth (Sahlberg et al., 2002; Gao et al., 2004; Partanen et al., 2004; Alaluusua and Lukinmaa, 2006). Its expression in skeletal tissue includes bone/cartilage progenitors and osteoclast precursors, as reviewed previously (Alhamad et al., 2022).

Polymorphisms in the *AHR* and *ARNT* genes in humans have been associated with increased risk of non-syndromic CP following environmental toxin exposure (Kayano et al., 2004). Additionally, polymorphisms in *AHRR*, which encodes the negative regulator of AhR, are associated with increased susceptibility to CP in human populations (Linnenkamp et al., 2020). Knockdown of *ahrra* and *ahrrb* in zebrafish, equivalent to human *AHRR*, also exacerbates TCDD-induced craniofacial defects, supporting the role of AhR repressors in protecting against excessive AhR signaling during craniofacial development (Jenny et al., 2009). AhR structure is a key determinant of strain-specific susceptibility to dioxins in mammals. C57BL/6J mice, carrying the high-affinity *Ah* ^*b*^ allele, exhibit a significantly higher susceptibility to TCDD-induced CP than DBA/2 mice, which express a low-affinity AhR variant (Moriguchi et al., 2003). Similarly, in rats, Long-Evans (L-E) rats have higher susceptibility to dioxins than the Han/Wistar (H/W) strain that expresses a highly resistant AhR variant (Huuskonen et al., 1994). Humanized AhR mice, which express the human form of AhR, demonstrated reduced sensitivity to TCDD-induced CP, consistent with the structural divergence of the human receptor from the murine forms (Moriguchi et al., 2003).

Loss of AhR abolishes the dioxin response: Ahr-null fetuses are resistant to TCDD-induced cleft palate and hydronephrosis, despite otherwise near-normal craniofacial development (Fernandez-Salguero et al., 1996; Mimura et al., 1997; Peters et al., 1999). Ahr-null mice also fail to induce genes encoding xenobiotic-metabolizing enzymes after dioxin exposure and show reduced liver size and diminished lymphocyte accumulation, indicating a physiological requirement for AhR in hepatic and immune development (Fernandez-Salguero et al., 1995). A constitutively active AhR (CA-AhR) transgenic mouse model has been developed to simulate sustained, low-level and ligand-independent AhR activation (Andersson et al., 2002). In these mice, a lymphoid-specific SRα promoter drives expression of a constitutively nuclear form of AhR, resulting in activation of AhR target genes independent of exogenous ligands in lymphocyte lineages. This model exhibits osteoclast overactivation leading to increased trabecular bone area and decreased cortical bone mineral density in female mice, which indicates that AhR activation in lymphocytes is sufficient to disrupt bone homeostasis (Bodrug et al., 1994; Andersson et al., 2002; Wejheden et al., 2010). However, constitutively active models specifically targeting osteoblasts, the developing palate, or teeth have not yet been reported. Together, these complementary loss- and gain-of-function models establish AhR as the key regulator of dioxin-induced developmental toxicity. While AhR signaling acts as an indispensable mediator during TCDD-induced CP, maternal AhR may play a protective role. A study by Thomae et al. demonstrated that embryos from *Ahr* ^−/−^ dams were significantly more sensitive to TCDD toxicity than those from *Ahr* ^+/+^ dams. When treated with TCDD, *Ahr* ^−/−^ dams produced embryos with a five-fold increase in CP incidence (Thomae et al., 2004). In another study, Hassoun et al. used reciprocal blastocyst transfer between TCDD-sensitive NMRI and non-sensitive DBA mouse strains to assess embryonic (zygotic) effects versus maternal influences (Hassoun et al., 1984). Nearly all NMRI fetuses developed CP in response to TCDD regardless of gestation in their own strain or DBA dams. Specifically, 100% of NMRI fetuses transferred to DBA mothers, and 85%–93% of NMRI fetuses in NMRI dams exhibited CP following TCDD exposure. In contrast, none of the DBA fetuses developed CP in the same conditions, irrespective of the maternal environment. Thomae’s design isolates the maternal genotype, whereas Hassoun’s blastocyst-transfer design isolates the embryonic (zygotic) genotype; together, the two studies suggest both compartments contribute, with the embryonic AhR predominating.

Smoking is another environmental activator of AhR signaling (Kasai et al., 2006; Kitamura and Kasai, 2007). Cigarette smoke constitutes a complex mixture of over 4,000 compounds, with dioxins ranking among its most biologically potent constituents, and both mainstream and sidestream smoke elicit robust AhR-mediated responses (Muto and Takizawa, 1989; Dertinger et al., 2001; Kasai et al., 2006; Kitamura and Kasai, 2007; Wilson et al., 2008). This mechanistic link is consistent with epidemiological evidence that maternal smoking during pregnancy is associated with an elevated incidence of orofacial clefts, including cleft palate (Fell et al., 2022), and offers a biologically plausible connection between smoke-derived dioxin exposure and human craniofacial defects.

### 3.2 Cellular and molecular mechanisms

TCDD affects multiple stages of palatogenesis in mice, including shelf growth, elevation, fusion, and post-fusion stability. One of the earliest outcomes of TCDD exposure is the inhibition of mesenchymal cell proliferation. At E13.5–15.5, mesenchymal cell proliferation declines significantly in TCDD-treated embryos compared to controls, particularly in the posterior palatal regions (Yamada et al., 2014; Sakuma et al., 2022; Zhang et al., 2024a).

TCDD exposure also alters the epigenetic landscape of the developing palate. It alters DNA methylation, including increased *Dnmt* expression and global hypermethylation as well as changes at *Tgf-β2/3* promoters (Wang et al., 2017; Zhang et al., 2018; Chen et al., 2021) and dysregulates non-coding RNAs such as lncRNA Meg3 and miR-214-3p (He et al., 2021; Dong et al., 2024; Liu et al., 2024). These modifications converge on the TGF-β/Smad signaling axis which regulates both palatal epithelium and mesenchyme (Abbott et al., 1992; Puga et al., 2005; Iwata et al., 2011; Pelikan et al., 2013).

TCDD exposure also induces apoptosis, as indicated by elevated expression of pro-apoptotic markers Bax and Caspase-3 in TCDD-treated palatal mesenchyme and a significant increase in TUNEL-positive apoptotic cells (Tao et al., 2020; Chen et al., 2022). This increased apoptosis is particularly pronounced in the posterior palatal mesenchyme, which may be another reason why clefts are often more severe in the posterior region of TCDD-exposed embryos (Yamada et al., 2014; Chen et al., 2020; Sakuma et al., 2022). TCDD suppresses autophagy by downregulating LC3-II, Atg5, and Beclin1 levels, leading to impaired cellular homeostasis and reduced mesenchymal viability (Zhang et al., 2024a). Simultaneously, the AKT/mTOR pathway is aberrantly activated, inhibiting autophagy and shifting cells toward a dysfunctional state that fails to support proper tissue growth. This imbalance in cellular metabolism and survival pathways results in hypoplastic palatal shelves in TCDD-exposed embryos (Zhang et al., 2024a). TCDD also activates the Rad54b DNA damage repair pathway, with mesenchymal cells exhibiting increased γ-H2AX expression, ultimately leading to apoptosis (Qiao et al., 2021).

TCDD alters the differentiation potential of palatal mesenchymal cells, particularly in the osteogenic lineages. TCDD exposure suppresses the expression of key osteogenic markers, including Runx2 and Osteopontin, in the developing palatal bone, with a more remarkable decline in the posterior regions (Yamada et al., 2014). The inhibition of osteogenic differentiation by TCDD was also documented *in vitro* in human fetal palatal mesenchymal cells (Liu et al., 2020). Additionally, TCDD disrupts myogenesis in the mesenchyme-guided myogenic lineages, causing a significant reduction in the expression of MyoD and Desmin in palatal muscles (Yamada et al., 2014).

TCDD exposure significantly disrupts the elevation of palatal shelves. In TCDD-treated embryos, palatal shelves frequently remained vertically-positioned along the sides of the tongue instead of transitioning to a horizontal orientation, resulting in delayed or failed fusion (Hu et al., 2015). The inability of the shelves to reposition has been associated with altered extracellular matrix composition and dysregulated Wnt signaling, a pathway that regulates cellular proliferation and cytoskeletal organization during palatal elevation (Yuan et al., 2012; Hu et al., 2015; Li et al., 2017; Reynolds et al., 2019). In TCDD-treated palatal shelves, both canonical Wnt/β-catenin and non-canonical Wnt5a signaling are disrupted (Hu et al., 2015). TCDD exposure also altered extracellular matrix composition in palatal shelves, with decreased levels of fibronectin and tenascin, which leads to impaired shelf flexibility and movement (Yuan et al., 2012).

TCDD disrupts epithelial remodeling, which is critical for the final stage of palatal fusion. An abnormal persistence of the medial edge epithelium (MEE) is observed in TCDD-treated embryos, where MEE failed to undergo programmed cell death and epithelial-mesenchymal transition (EMT), two processes that are essential for the mesenchymal continuity in the fused palate (Gao et al., 2019; Chen et al., 2020; Chen et al., 2022). TGF-β signaling plays a central role in epithelial-mesenchymal interactions, extracellular matrix remodeling, and palatal fusion (Gan et al., 2009; Gao et al., 2019; Chen et al., 2021; Won et al., 2023). TGF-β3, in particular, is essential for MEE EMT and degradation, and its suppression leads to CP. TCDD exposure significantly suppressed TGF-β3 expression in the developing palate, preventing EMT, while exogenous TGF-β3 supplementation in palatal organ cultures partially restored palatal fusion (Thomae et al., 2005; Gao et al., 2019; Chen et al., 2021). TCDD-exposed embryos frequently exhibit anterior palatal fusion but persistent clefts in the posterior regions (Sakuma et al., 2018). Histological analyses revealed that these cleft regions exhibited sparse mesenchymal cell populations, defective epithelial continuity, and basement membrane fragmentation. There were also cases where initial fusion occurred but post-fusion defects developed (Fujiwara et al., 2008). The expression of E-cadherin, α-catenin, and β-catenin was significantly reduced in the midline epithelial seam of TCDD-exposed embryos, suggesting compromised cell-cell adhesion that may lead to fusion instability (Fujiwara et al., 2008; Imura et al., 2010; Sakuma et al., 2018; Sakuma et al., 2022). Furthermore, a reduction in laminin and collagen IV deposition, both essential components of the basement membrane, may contribute to structural instability after fusion (Fujiwara et al., 2008; Sakuma et al., 2018).

### 3.3 Pharmacological and nutritional interventions

Several pharmacological/nutritional interventions have been explored to alleviate TCDD-induced CP, targeting AhR activation, oxidative stress, or other signaling pathways. AhR antagonists, such as resveratrol (Jang et al., 2008) and α-naphthoflavone (Jang et al., 2007; Yuan et al., 2017), significantly reduce the incidence of CP in TCDD-exposed mice. Daily administration of quercetin, a flavonoid with antioxidant and anti-inflammatory properties, leads to a statistically significant decrease in CP frequency compared to TCDD exposure alone (Satake et al., 2022). Additionally, quercetin restores mesenchymal proliferation and maintains epithelial integrity in TCDD-exposed palatal shelves (Satake et al., 2022). Other antioxidants, such as vitamin E succinate and ellagic acid, have been efficient in reducing oxidative stress and TCDD-induced fetal growth retardation but failed to fully prevent CP formation (Hassoun et al., 1997). In humans, folic acid (FA) supplementation has been explored as a protective strategy against CP, and maternal supplementation in early pregnancy indeed reduced the risk of CP among multiple studies (Jahanbin et al., 2018; Xu et al., 2018a). In mice, FA administration at 10 mg/kg reduced TCDD-induced CP incidence from 92.9% to 73.1%, while 5 mg/kg and 15 mg/kg doses showed fewer protective effects (86.0% and 84.0% CP incidence, respectively). FA treatment also partially preserved MEE integrity, showing smooth and fully expanded epithelial surfaces with visible filopodia-like structures, although still fewer and shorter compared to controls (Yuan et al., 2017). In contrast, vitamin B12 supplementation failed to prevent CP in TCDD-exposed embryos, despite a partial modulation of the TGF-β pathway (Zhao et al., 2014). These findings, by indicating that antioxidants only partially mitigate TCDD-induced defects, whereas AhR antagonists are more effective, suggest that oxidative stress might be a downstream effector of AhR-driven transcriptional responses rather than the primary inducer. Hence, scavenging reactive oxygen species alone provides incomplete protection.

### 3.4 Interactions with signaling pathways

Molecular interactions between TCDD and other signaling pathways also regulate the occurrence of CP. Retinoic acid (RA) plays a crucial role in craniofacial development, and various studies have proven that its disruption results in CP (Abbott and Birnbaum, 1989; Abbott et al., 1989; Padmanabhan and Ahmed, 1997). TCDD inhibits RA-induced gene expression in murine embryonic palatal mesenchymal cells, suppressing transcription of the retinoic acid receptor β (RARβ) and the type II cellular retinoic acid binding protein (CRABP-II) without affecting RA binding to receptors, suggesting that TCDD interferes with downstream signaling rather than through direct receptor interaction (Weston et al., 1995). Paradoxically, TCDD requires an intact RA pathway to induce CP, as demonstrated in mouse models where RA-deficient embryos were resistant to TCDD toxicity (Jacobs et al., 2011). Proteomic analysis also suggested convergent molecular mechanisms between TCDD and RA exposure in mouse palatal tissue in the occurrence of CP (Wang et al., 2019). These findings indicate that while TCDD disrupts certain levels of RA signaling, it simultaneously requires baseline RA activity to induce CP. Furthermore, EGF signaling plays a modulatory role in TCDD-induced CP, influencing tissue-specific sensitivity (Abbott et al., 2003; Abbott et al., 2005). Pax3- and Pax7-deficient mice also exhibit increased susceptibility to TCDD-induced craniofacial malformations, including bifid palates and frontonasal clefts. These phenotypes are linked to upregulation of AhR and reduced proliferation of Sox9^+^ CNCCs. In these cases, treatment with an AhR antagonist rescues facial defects, suggesting Pax3/7 as transcriptional regulators safeguarding against AhR-mediated craniofacial defects (Zalc et al., 2015).

## 4 Effects of dioxin on craniofacial bone development

Craniofacial bone development is a complex process involving both CNCCs and mesodermal cells to form the diverse facial and cranial structures, including the palatine bone (Stimson and Jones, 2023). We have focused on the formation of the palate and the palatine bone in the preceding section. Increasing evidence from vertebrate models indicates that the skull is particularly sensitive to dioxin toxicity. Across animal models, including mouse, rat, and zebrafish, exposure can disrupt the size, shape, composition, and histological integrity of the developing bones (Keller et al., 2008; Burns et al., 2015; Sholts et al., 2015a; Sholts et al., 2015b; Staal et al., 2018; Wang et al., 2025).

### 4.1 Structural consequences across species

Dioxin exposure induces a wide array of structural alterations in craniofacial bones, with effects observed across multiple animal models. In mice, embryos exposed to TCDD at E12.5 exhibit premature fusion of metopic and coronal sutures, shortened palatal processes of the maxilla and palatine bones, and outward displacement of the pterygoid processes (Wang et al., 2025). Frontal bone length, maxillary width, and mandibular length are all significantly reduced by TCDD (Wang et al., 2025). Quantitative analyses also reveal strain-specific changes in mandibular shape following prenatal TCDD exposure, with effects more pronounced in sensitive mouse strains (Allen and Leamy, 2001; Keller et al., 2008). TCDD in rats leads to reduced mineral content and altered geometry of cranial bone, with strain-specific differences attributed to AhR structure and activity (Finnilä et al., 2010; Herlin et al., 2010). In rats, *in utero*, lactational, and adult exposure to TCDD all cause reductions in craniofacial bone size and dose-dependent changes in shape, particularly in females, with alterations localized to cranial suture regions and anterior facial structures (Sholts et al., 2015b). Evidence from chick embryos shows that TCDD reduces levels of calcium, magnesium, manganese, and zinc in the calvaria without gross morphological changes, indicating early mineralization defects (Dobrzynski et al., 2019). In zebrafish, embryonic exposure to TCDD results in reduced ossification of dermal bones and defects analogous to CP in mammals (Burns et al., 2015). These structural defects are accompanied by reductions in craniofacial length and head-trunk angle, which persist across developmental stages and can extend into the F1 generation, suggesting long-lasting or heritable impacts of early TCDD exposure (Meyer et al., 2024). Early TCDD exposure disrupts *sox10* ^+^ neural crest-derived chondrocytes in zebrafish, which results in decreased *sox10* expression in ventral jaw structures and neurocranium, cleft-like malformations of the ethmoid plate, and reduced collagen type II deposition, indicating impaired chondrocyte maturation (Cintrón-Rivera et al., 2023). TCDD also targets *tcf21* ^+^ mesodermal progenitors in zebrafish, leading to disrupted jaw muscle fiber formation, cranial nerve compression, and loss of major arteries such as the ventral aorta, revealing a multi-lineage failure during craniofacial development (Cintrón-Rivera et al., 2023). Among PCB congeners tested in zebrafish embryos, PCB126 is the most toxic and causes craniofacial malformations in a concentration- and congener-dependent manner. (Sişman et al., 2007). PCB126 disrupts neural crest-derived branchial cartilage formation, and notably, early neural crest migration and patterning remain unaffected while the cartilages fail to grow to normal size (Grimes et al., 2008).

### 4.2 Cellular and molecular mechanisms

TCDD inhibits osteogenic differentiation in mammalian cell culture systems (Carpi et al., 2009; Yun et al., 2018). TCDD disrupts adhesion and migration in osteoblast-like MG-63 cells, by downregulating integrin-α5, E-cadherin, and CXCL12/CXCR4 signaling, alongside suppression of MAPK pathway activity, all of which are essential for functional osteoblast behavior and bone matrix formation (Yun et al., 2018). In MC3T3-E1 pre-osteoblast cultures, TCDD exposure increases oxidative stress and decreases cell viability and mitochondrial membrane potential, while impairing the expression of osteogenic markers such as alkaline phosphatase, osterix, osteocalcin, and bone sialoprotein (Choi et al., 2017; Suh et al., 2018). These effects are mitigated by natural AhR antagonists 27-deoxyactein and actein, which restore antioxidant defenses and osteogenic differentiation, suggesting that AhR mediates both oxidative stress and TCDD-induced osteotoxicity (Suh et al., 2018). In human bone-derived mesenchymal stem cells (hBMSCs), TCDD impairs osteoblast differentiation and mineralization while preserving stemness-associated markers such as SOX2, NANOG, and SALL4, suggesting an arrest at the progenitor stage (Watson et al., 2019). These effects are reversed by AhR inhibition with GNF351, suggesting that the suppression of osteogenic differentiation in hBMSCs is AhR-mediated (Watson et al., 2019). Moreover, transcriptional and structural instability can contribute to impaired osteoblast function as the expression of cytoskeletal proteins (e.g., vimentin, tropomyosins), calcium-binding proteins (e.g., calreticulin, annexin II), and nuclear scaffolding elements (e.g., lamin A) is disrupted in differentiating osteoblasts when exposed to TCDD (Carpi et al., 2009). This mechanism is conserved in other species. In zebrafish, *ahr2*-null mutants (analogous to *Ahr*-null mammals) are resistant to TCDD-induced toxicity and the associated downregulation of *sox9b*, a zebrafish paralog of *Sox9*, a critical transcription factor for chondrogenesis and osteogenesis (Xiong et al., 2008; Garcia et al., 2018). The induction of a long noncoding RNA that suppresses *sox9b* expression is disrupted in *ahr2* mutants, providing a direct molecular link between AhR and *sox9b* repression (Xiong et al., 2008). On the other hand, Foxq1b, a forkhead-box transcription factor expressed in the zebrafish jaw primordium, is robustly induced by TCDD in an Ahr2-dependent manner, and its upregulation spatially coincides with Meckel’s cartilage malformations (Planchart and Mattingly, 2010). Forkhead-box factors act as competency factors for facial cartilage specification, and Foxc1 increases enhancer accessibility for Sox9 to activate the cartilage-specific transcriptome (Xu et al., 2018b; Xu et al., 2021). As such, TCDD-induced dysregulation of Fox signaling may perturb transcriptional and epigenetic regulation during facial cartilage formation.

In addition to direct effects on osteogenic differentiation, TCDD disrupts endocrine and paracrine signaling pathways essential for craniofacial bone formation. In neonatal mice, TCDD up-regulates renal vitamin D 1α-hydroxylase (Cyp27b1), which results in an approximately two-fold increase in the circulating active form of vitamin D, 1,25-dihydroxyvitamin D3, without changes in parathyroid hormone or serum calcium levels (Nishimura et al., 2009). Although elevated active vitamin D is generally associated with enhanced mineralization, in this setting, it was accompanied by suppression of osteogenesis with defective mineralization and thickened osteoid seams, while osteoclast activity remained unaffected (Nishimura et al., 2009). This phenomenon might be explained by the level at which TCDD acts. TCDD disrupts the vitamin D axis and directly inhibits osteoblast differentiation (Watson et al., 2019). This cell-autonomous inhibition overrides the pro-mineralization effect that elevated 1,25-dihydroxyvitamin D3 would otherwise have. Parallel findings show that TCDD alters RA homeostasis by repressing the RA–degrading enzyme *Cyp26a1* and *Cyp26b1* and increasing *Raldh3* expression while repressing late osteoblast markers (*Alp*, *Ocn*) (Herlin et al., 2021). These findings exemplify TCDD-induced asynchrony between differentiation and mineralization through crosstalk with vitamin D and RA signaling. Another endocrine input is estrogen signaling. TCDD in mice leads to a significant downregulation of *Runx2* and *Col1a1* in the palatal mesenchyme, as well as suppression of estrogen receptor α (Wang et al., 2025). Moreover, exposure of human fetal palatal mesenchymal cells to TCDD reduces cell proliferation, represses early and late osteogenic markers, and impairs matrix mineralization (Wang et al., 2025). Co-treatment with the AhR antagonist BAY2416964 or the ERα agonist PPT rescues gene expression and restores mineral deposition, indicating that TCDD-mediated inhibition of craniofacial osteogenesis operates through a dual AhR/ERα-dependent mechanism (Wang et al., 2025).

Furthermore, emerging evidence suggests sex-dependent susceptibility to dioxins. Dioxin-induced craniofacial bone-shape changes are more pronounced in females (Sholts et al., 2015b), and a constitutively active AhR produces a female-specific skeletal phenotype (Wejheden et al., 2010), which is in line with documented inhibitory crosstalk between AhR and ERα. TCDD-activated AhR/ARNT binds inhibitory XREs adjacent to estrogen response elements (EREs) in the promoters of estrogen-target genes to repress ERα-induced transcription of cathepsin D, c-fos, and HSP27 in MCF-7 breast cancer cells (Safe et al., 2000). Ligand binding also stimulates AhR assembly into a CUL4B ubiquitin ligase complex that directly targets ERα for ubiquitination and proteasomal degradation (Ohtake et al., 2007).

Direct human data on dioxin effects on craniofacial bone remain scarce, and targeted human studies are therefore needed to confirm the translational relevance of the animal findings summarized here.

## 5 Effects of dioxin on odontogenesis and teeth

**FIGURE 4:**
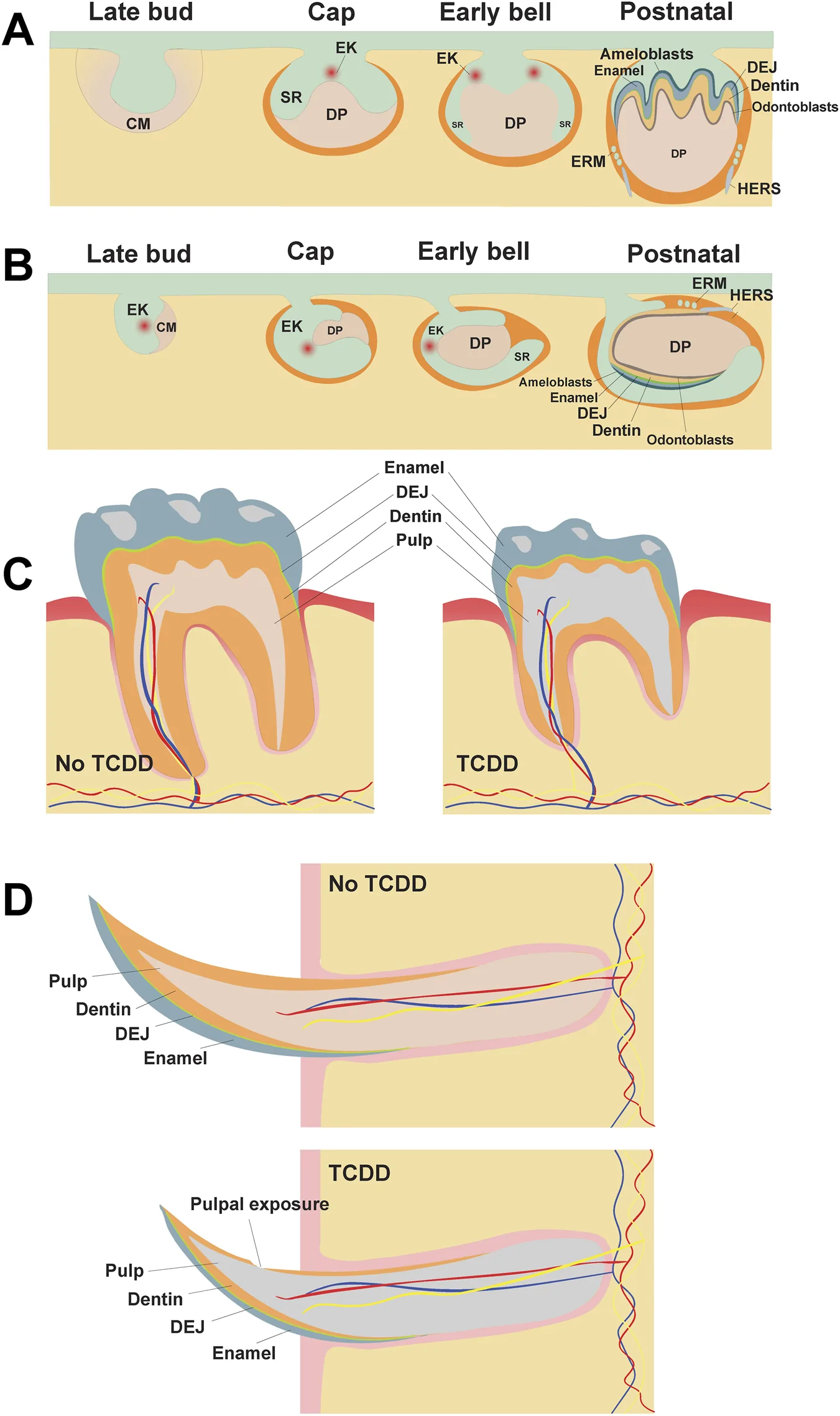
TCDD-induced developmental and structural defects in murine teeth. **(A,B)** Representative cross-sectional views of normal tooth development showing progressive stages of odontogenesis with distinct tissue layers, for the molar **(A)** and incisor **(B)**, from the late bud stage through the cap and early bell stages to the postnatal tooth. Labeled structures include the enamel knot (EK), condensing mesenchyme (CM), stellate reticulum (SR), dental papilla (DP), ameloblasts, enamel, DEJ, dentin, odontoblasts, epithelial rests of Malassez (ERM), and Hertwig’s epithelial root sheath (HERS). **(C)** Comparative views of erupted molar from control (No TCDD, left) and TCDD-exposed (right) mice at postnatal stages. Control molars display normal architecture with well-defined enamel, dentin, DEJ, and the pulp chamber. TCDD-exposed molars exhibit structural abnormalities, including reduced enamel, an enlarged pulp chamber with thinner surrounding dentin, and altered root morphology. **(D)** Higher-magnification comparative views of the incisor crown margin from control (upper panel) and TCDD-exposed (lower panel) mice. Control teeth display normal architecture with well-defined enamel (outer layer), dentin (middle layer), dentinoenamel junction (DEJ), and pulp chamber. TCDD-exposed teeth exhibit structural abnormalities, including reduced enamel, thinner dentin, and pulpal exposure. Anatomical structures are labeled: enamel (outermost mineralized layer), dentin (bulk mineralized tissue), DEJ (interface between enamel and dentin), and pulp (central vascularized tissue).

Tooth formation, also known as odontogenesis, is a complex process that begins in the embryonic stage and continues into early childhood. It involves the interaction of oral epithelial and mesenchymal cells, leading to the development of dental structures, a process that is divided into the initiation, bud, cap, bell, and maturation stages (Svandova et al., 2020). During initiation, the dental lamina forms and grows into a small bud shape, giving rise to the tooth bud. The bud grows under the influence of the surrounding mesenchyme and develops a concave cap shape, which forms the enamel organ. The surrounding condensed mesenchyme cells give rise to the dental papilla and dental follicle, which later form the pulp and periodontal ligament, respectively. The bell stage shapes the tooth while allowing ameloblast and odontoblast differentiation. Finally, in the maturation stage, these tissues mineralize, completing odontogenesis (Figure 4).

Dioxins affect both developing (Partanen et al., 1998; Kattainen et al., 2001; Keller et al., 2007) and developed teeth (Miettinen et al., 2006). In human populations, studies have reported the association of dioxins with dental issues, including early tooth eruption (Alaluusua et al., 1996; Alaluusua et al., 2004) and natal teeth at birth (Miller, 1985; Yamashita and Hayashi, 1985). In the 1979 Yu-Cheng incident in Taiwan, in which mothers were contaminated with PCBs and PCDF, children exposed *in utero* showed a generalized ectodermal-tissue disorder that included natal teeth and abnormalities of the gingiva, nails, and skin, consistent with dioxin-like disruption of odontogenesis and other ectodermal derivatives (Rogan et al., 1988). While these findings suggest that dioxins might disrupt normal tooth development in humans (Takiguchi et al., 2022), animal studies provide a more comprehensive mechanistic understanding.

### 5.1 Time- and dose-dependent effects

In rodents, odontogenesis begins *in utero* and continues postnatally. Tooth formation in mice initiates around gestational day 11 (GD11) with the dental lamina, followed by the bud stage at GD12–GD13, the cap stage by GD14–GD15, and the bell stage around GD16 (Catón and Tucker, 2009). Crown formation and mineralization begin perinatally, and root formation occurs postnatally, with eruption beginning around postnatal day (PND)14 (Catón and Tucker, 2009). Rats follow a similar developmental timeline but are slightly delayed by about one to two days (Catón and Tucker, 2009). TCDD affects tooth development with a strong time-dependent relationship, where exposure in earlier developmental stages leads to more severe defects, and later exposure affects mineralization and eruption. The earliest stages of tooth development, particularly the initiation and bud stages, are the most vulnerable, with GD11 exposure in rats causing the highest frequency of missing third molars (Miettinen et al., 2002). Similarly, exposure at GD13 in mice resulted in complete third molar agenesis in certain strains (Keller et al., 2007). Later exposure at GD15 led to a progressive reduction in molar size and increased caries susceptibility, suggesting that TCDD interferes with both morphogenesis and mineralization (Kattainen et al., 2001; Miettinen et al., 2006). Postnatal exposure caused significant defects, but only at higher concentrations; for example, a single dose of 50 or 1000 μg/kg at PND1 led to delayed eruption, incomplete calcification, and structural abnormalities in molars (Lukinmaa et al., 2001). The degree of disruption was highly time-dependent, as early postnatal exposure impaired root formation and delayed eruption, whereas later exposure primarily disturbed enamel maturation (Gao et al., 2004). Moreover, postnatal TCDD exposure in rats led to pronounced defects in both incisors and molars, including enamel thinning, reduced mineral density, malformed crowns and roots, altered incisor curvature and torsion, changed molar geometry and mineralization, and reduced odontoblast numbers (Dobrzyński et al., 2022). While antioxidant co-treatment with acetylsalicylic acid or vitamin E partially preserved mineral composition and reduced inflammation, neither agent fully prevented the structural defects (Dobrzyński et al., 2022). This temporal sensitivity suggests that TCDD affects odontogenesis depending on the stage, with early exposure leading to developmental failure and later exposure impairing differentiation and mineralization.

The severity of TCDD’s effects also follows a dose-dependent pattern, where lower doses primarily reduce molar size and delay mineralization, while higher doses result in complete agenesis and structural deformities (Kattainen et al., 2001; Lukinmaa et al., 2001; Gao et al., 2004; Miettinen et al., 2006; Keller et al., 2007). Maternal exposure at a lower dose (0.03–1.0 μg/kg) caused reductions in third molar size, with complete third molar agenesis observed at 1.0 μg/kg in sensitive strains (Kattainen et al., 2001; Miettinen et al., 2006). In mice, the first molar cusp morphology was altered at doses as low as 0.01 μg/kg, indicating that even a low dose of TCDD exposure can disrupt early patterning in sensitive strains (Keller et al., 2007). Higher doses (50–1000 μg/kg) had progressively more severe effects, with postnatal exposure to higher doses leading to arrested molar root formation, pulpal necrosis, and mineralization defects (Lukinmaa et al., 2001; Gao et al., 2004). Chronic exposure over a 20-week period (0.17–170 μg/kg) resulted in dose-responsive pulpal perforation and color defects in incisors, with no significant differences between resistant and susceptible rat strains at the highest doses, suggesting that high concentrations of TCDD override genetic variations (Kiukkonen et al., 2002). The correlation between missing maxillary and mandibular third molars (phi coefficient = 0.86) suggests a predictable, dose-related pattern in TCDD-induced third molar agenesis (Keller et al., 2007). Varying sensitivity to TCDD is exhibited in different tooth types, with third molars being the most affected, followed by second and first molars, while incisors show a distinct phenotype due to their continuous growth in rats and mice. Third molars were most susceptible, likely due to their later initiation in development, making them highly vulnerable to disruptions in early signaling pathways (Miettinen et al., 2002; Keller et al., 2007). Second and first molars exhibited more subtle effects, such as reduced size and altered cusp morphology, but were rarely completely absent (Kattainen et al., 2001; Keller et al., 2007). Incisors, in contrast, exhibited significant structural defects under chronic or high-dose exposure, with thinner enamel and dentin, pulpal perforation, and failure of secondary dentin formation (Kiukkonen et al., 2002; Alaluusua et al., 2004).

### 5.2 Tissue-specific effects: enamel, dentin, and periodontium

TCDD exposure affects different tooth structures distinctively, with dentin formation being disrupted at lower doses, while enamel maturation is more sensitive to later exposure. In postnatal studies, TCDD exposure led to thickened predentin layers, with reduced mineralization and an irregular boundary between predentin and mineralized dentin, indicating a failure in odontoblast differentiation (Lukinmaa et al., 2001; Gao et al., 2004). At higher doses, pulp necrosis and pulpal perforation were common, further impairing dentin formation (Alaluusua et al., 1993; Lukinmaa et al., 2001). Enamel, on the other hand, exhibited pronounced matrix retention defects, particularly when exposure occurred during the late postnatal period, suggesting that ameloblast function was directly disrupted by TCDD exposure (Gao et al., 2004). The impaired enamel maturation led to increased caries susceptibility, particularly in second and third molars, with higher doses leading to deeper lesions that extended into the dentin (Miettinen et al., 2006). This increased caries risk may be due to both direct defects in enamel mineralization and indirect effects such as altered salivary gland function or immune suppression, which could affect oral bacterial colonization (Miettinen et al., 2006). The periodontal ligament (PDL) and pulp tissues were also affected, particularly at higher doses, where PDL necrosis, pulp necrosis, and perforation were observed (Lukinmaa et al., 2001). PDL development was less frequently examined, but existing studies suggest that TCDD exposure leads to PDL necrosis (Kiukkonen et al., 2002). Exposure to AhR agonists such as β-naphthoflavone in mink induces squamous epithelial proliferation, gingival overgrowth, and underlying osteolysis in the mandible and maxilla, closely resembling lesions caused by TCDD (Ellick et al., 2013; Matz et al., 2019). These lesions appear to form from epithelial rests of Malassez, clusters of epithelial cells in the periodontal ligament matrix surrounding the tooth root, and result in tooth loosening and alveolar bone resorption (Matz et al., 2019).

### 5.3 Molecular mechanisms and genetic susceptibility

The effects of TCDD on tooth development are mainly mediated through the AhR pathway. Prolonged TCDD exposure leads to a paradoxical suppression of AhR and Cyp1a1 expression in dental tissues, particularly in ameloblasts and odontoblasts, potentially through negative feedback mechanisms (Gao et al., 2004). High-dose exposure (1000 μg/kg) nearly abolishes AhR and Cyp1a1 protein levels in ameloblasts and odontoblasts while maintaining expression in the papillary layer. This phenomenon may indicate that ameloblasts and odontoblasts are more sensitive to TCDD than the papillary layer, and TCDD likely interferes with ameloblast and odontoblast differentiation (Alaluusua et al., 1993; Gao et al., 2004; Miettinen et al., 2006). The timing of AhR suppression corresponds with key developmental events in enamel and dentin formation, suggesting that TCDD’s toxic effects might be mediated through dysregulation of AhR-responsive genes that control cellular differentiation and matrix mineralization. Beyond AhR, additional genetic factors affect susceptibility to TCDD-induced dental defects, as CBA/J and C3H/HeJ mice have higher rates of third molar agenesis when compared to other strains with the same high-affinity AhR variant (Keller et al., 2007).

## 6 Conclusion

**FIGURE 5:**
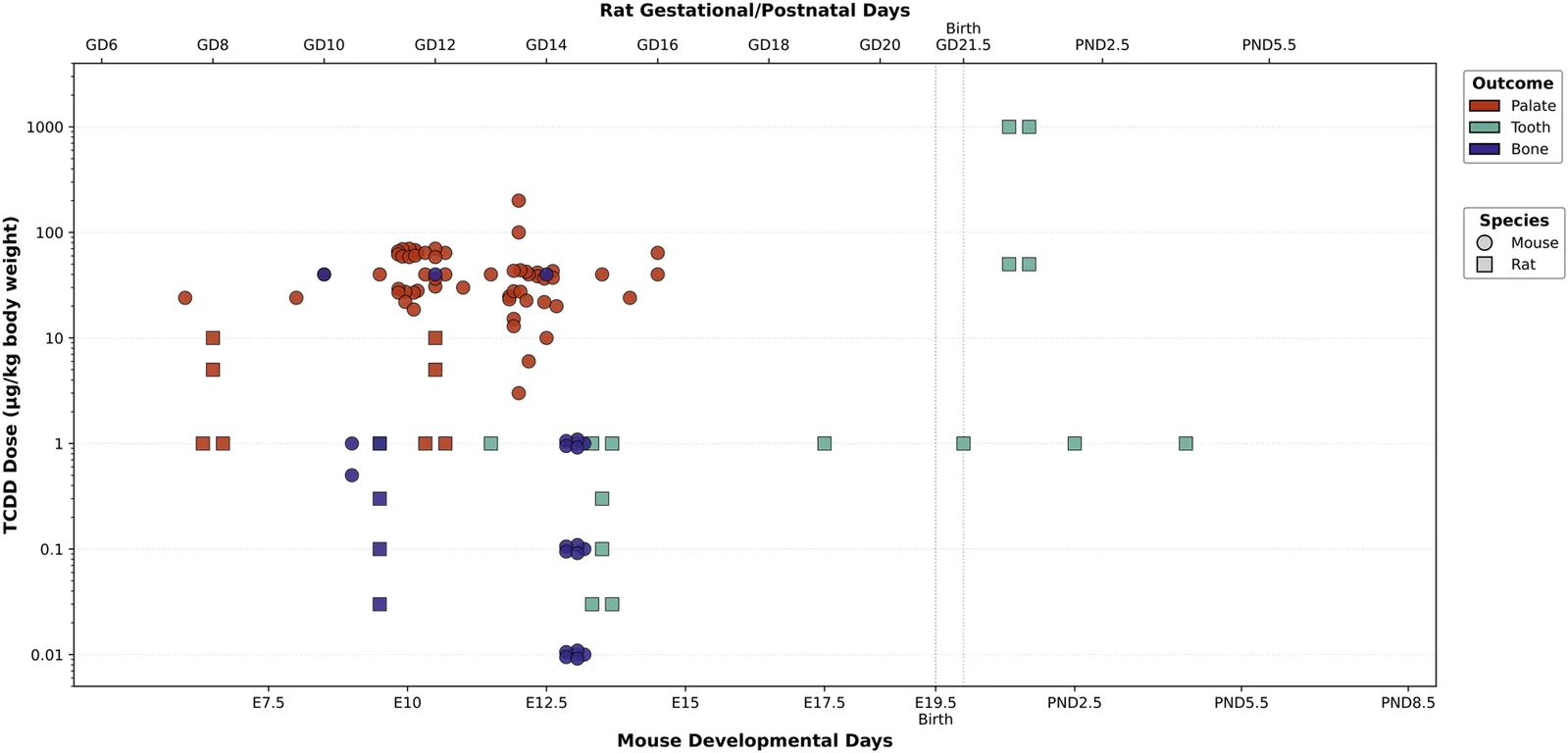
TCDD dose and developmental day across *in vivo* rodent studies of craniofacial and dental development. Each marker represents one experimental condition (species × strain × dose × exposure-day) explicitly reported in the cited primary literature. Y-axis: TCDD dose (μg/kg body weight, log scale). X-axis: developmental day, with mouse (E/PND) on the bottom and rat (GD/PND) on the top; vertical dashed lines indicate birth. Markers: ● mouse, ■ rat; colors: red = palatogenesis (cleft palate), green = odontogenesis (dental defects), blue = craniofacial bone development. Studies reporting only dose ranges, *in vitro* experiments, non-rodent models, or those without an explicit dose × day pair were excluded; chronic-dosing and systemic-bone cohorts are tabulated separately. *n* = 107 observations/36 studies (79 mouse; 28 rat). The compilation includes additional *in vivo* rodent studies not otherwise cited in this review (Abbott and Birnbaum, 1990; Couture et al., 1990; Abbott et al., 1999; Takagi et al., 2000; Dragin et al., 2006; Li et al., 2010; Yuan et al., 2016; Gao et al., 2017; Gao et al., 2020; Wang et al., 2020; Gao et al., 2023; Yongkai et al., 2024; Yu et al., 2024).

The evidence presented in this review demonstrates that dioxin exposure causes widespread and lasting effects on dental and craniofacial development. Dioxins affect multiple developmental systems, including the teeth, palate, and craniofacial bones, and their effects are mediated by a combination of disrupted molecular signaling pathways, including but not limited to the AhR pathway. The severity and incidence of these defects are highly dependent on the dose, timing of exposure, and genetic background, with early developmental windows showing higher vulnerability (Figure 5). Moreover, the persistence of dioxins in the environment and their accumulation in biological tissues underscore their relevance as enduring public health hazards.

In summary, *in vivo* studies in mice, rats, zebrafish, and chick defined the phenotypes and their dependence on dose, exposure timing, and genetic background: cleft palate, reduced ossification and altered mineral content of cranial bone, premature sutural fusion and shortened palatal processes, and stage-dependent dental defects including third molar agenesis, enamel hypoplasia, dentin defects, and increased caries susceptibility. These models also established AhR as the main mediator of dioxin-induced developmental defects and demonstrated the crosstalk of the AhR pathway, vitamin D, TGF-β, and Wnt signaling. On the other hand, *in vitro* studies in human fetal palatal mesenchymal cells, osteoblast-like cells, pre-osteoblasts, and human bone-derived mesenchymal stem cells focused on the cellular basis of these effects, showing that TCDD suppresses osteogenic differentiation and mineralization, disrupts osteoblast adhesion and migration, arrests cells at the progenitor stage, and increases oxidative stress.

Although direct human cohort data on dioxin-induced craniofacial defects remain limited, the strong body of evidence from animal and human cell-culture models supports continued regulatory monitoring and reductions in dioxin emissions. Second, further research is needed to clarify mechanisms of variant-specific susceptibility, especially in human populations, where genetic polymorphisms in AhR-related genes may modulate risk. Third, mechanistic studies should continue to investigate how dioxin exposure intersects with key developmental signaling pathways such as Wnt, TGF-β, and RA, with an emphasis on identifying potential targets for pharmacologic or nutritional intervention. Fourth, systematic comparisons across dioxin-like congeners are needed. Establishing whether individual congeners produce identical or subtly different developmental phenotypes would test the AhR-centered model directly, and it would improve the relevance of these findings to human exposures, which involve congener mixtures rather than TCDD alone. Finally, there is a need to explore protective strategies, including antioxidant and AhR antagonist therapies, which may offer partial mitigation of dioxin-induced teratogenic effects in vulnerable populations.
